# The rs9340799 polymorphism of the e*strogen receptor alpha (ESR1)* gene and its association with breast cancer susceptibility

**DOI:** 10.1038/s41598-021-97935-8

**Published:** 2021-09-20

**Authors:** Shing Cheng Tan, Teck Yew Low, Ezanee Azlina Mohamad Hanif, Mohamad Ayub Khan Sharzehan, Hamed Kord-Varkaneh, Md Asiful Islam

**Affiliations:** 1 UKM Medical Molecular Biology Institute, Universiti Kebangsaan Malaysia, Kuala Lumpur, Malaysia; 2 Department of Clinical Nutrition and Dietetics, Student Research Committee, Faculty of Nutrition and Food Technology, Shahid Beheshti University of Medical Sciences, Tehran, Iran; 3 Department of Haematology, School of Medical Sciences, Universiti Sains Malaysia, Kubang Kerian, Kelantan, Malaysia

**Keywords:** Cancer, Genetics, Breast cancer, Diagnostic markers, Predictive markers, Breast cancer, Cancer genetics, Tumour biomarkers

## Abstract

The *ESR1* rs9340799 polymorphism has been frequently investigated with regard to its association with breast cancer (BC) susceptibility, but the findings have been inconclusive. In this work, we aimed to address the inconsistencies in study findings by performing a systematic review and meta-analysis. Eligible studies were identified from the Web of Science, PubMed, Scopus, China National Knowledge Infrastructure, VIP and Wanfang databases based on the predefined inclusion and exclusion criteria. The pooled odds ratio (OR) was then calculated under five genetic models: homozygous (GG vs. AA), heterozygous (AG vs. AA), dominant (AG + GG vs. AA), recessive (GG vs. AA + AG) and allele (G vs. A). Combined results from 23 studies involving 34,721 subjects indicated a lack of significant association between the polymorphism and BC susceptibility (homozygous model, OR = 1.045, 95% CI 0.887–1.231, P = 0.601; heterozygous model, OR = 0.941, 95% CI 0.861–1.030, P = 0.186; dominant model, OR = 0.957, 95% CI 0.875–1.045, P = 0.327; recessive model, OR = 1.053, 95% CI 0.908–1.222, P = 0.495; allele model, OR = 0.987, 95% CI 0.919–1.059, P = 0.709). Subgroup analyses by ethnicity, menopausal status and study quality also revealed no statistically significant association (P > 0.05). In conclusion, our results showed that the *ESR1* rs9340799 polymorphism was not associated with BC susceptibility, suggesting its limited potential as a genetic marker for BC.

## Introduction

According to the World Health Organization statistics, breast cancer (BC) is the most common malignant tumor type, as well as a leading cause of mortality in the female population^1,2^. Like other malignancies, risk factors of BC are primarily genetic predisposition and environmental influences^3^. It has been reported that genetic background or familial history accounts for ~ 20–25% of overall BC incidence^4^. Among the ~ 80 genetic loci known to be associated with susceptibility to BC, the *BRCA1* and *BRCA2* loci carry the highest risk^5,6^. Together with other high- and medium-penetrance germline mutations located at the loci of *TP53*, *PTEN*, *ATM*, *BRIP1*, *CHEK2* and *PALB2*, they made up ~ 15–20% of the genetic risk of BC^7–9^. Common low‑penetrance genetic polymorphisms account for the remaining risk for BC susceptibility^10,11^. On the other hand, environmental and lifestyle risk factors for BC include the consumption of oral contraceptive, cigarette smoking, alcohol consumption, breastfeeding and delayed age at first childbirth^12–14^.

Among these risk factors, it has been specifically pointed out that estrogen can act as a carcinogen, not only by causing chromosome segregation errors as well as structural chromosomal alterations, but also by stimulating the uncontrolled proliferation of mutated breast cells^4,15,16^. Mounting evidence from population-based studies has corroborated the association of endogenous and exogenous circulating estrogen in BC etiology and the increased risk of BC in premenopausal women^17^. The physiological receptors for estrogen are estrogen receptors (ER), which function to mediate the effect of estrogen on breast cells. Binding of estrogen to ER promotes the growth and differentiation of the normal breast cells and can lead to breast carcinogenesis^18^. There are two ER isoforms, i.e., ERα and ERβ. These two ER isoforms are respectively encoded by two distinct genes, *ESR1* and *ESR2*^19^. ERα has a higher level of expression in the breast tissue between these two isoforms, hence it is frequently implicated in BC development^20^.

The focus of this meta-analysis is the ERα-encoding gene, *ESR1*, which is highly polymorphic. Among the many polymorphisms in *ESR1*, the two best-studied ones are rs2234693 (also known as PvuII or 397T>C) and rs9340799 (also known as XbaI or 351A>G) polymorphisms. Both polymorphisms are located in intron 1, respectively at 1,397 bp and 351 bp upstream of exon 2 of the gene, and have been associated with several female cancers, including BC and endometrial cancer^21–23^. However, the association of the two polymorphisms with BC susceptibility has been described with conflicting results in many studies^24–27^. To sort out this inconsistency, a meta-analysis on rs2234693 was performed in 2018, and showed that the polymorphism was significantly associated with a decreased BC susceptibility^28^. As for rs9340799, a meta-analysis based on seven previous studies was reported by Zhang et al. in 2015, and found no significant association between the polymorphism and BC susceptibility under all three genetic models examined, even when the data were stratified into subgroups according to the ethnicity and source of controls^29^. In this current work, we attempted to perform an updated meta-analysis on the relationship between *ESR1* rs9340799 polymorphism and BC susceptibility, by including a large number of additional studies that have been left out by Zhang et al. or have only been published after 2015.

## Results

### Study selection and characteristics

**Figure 1:**
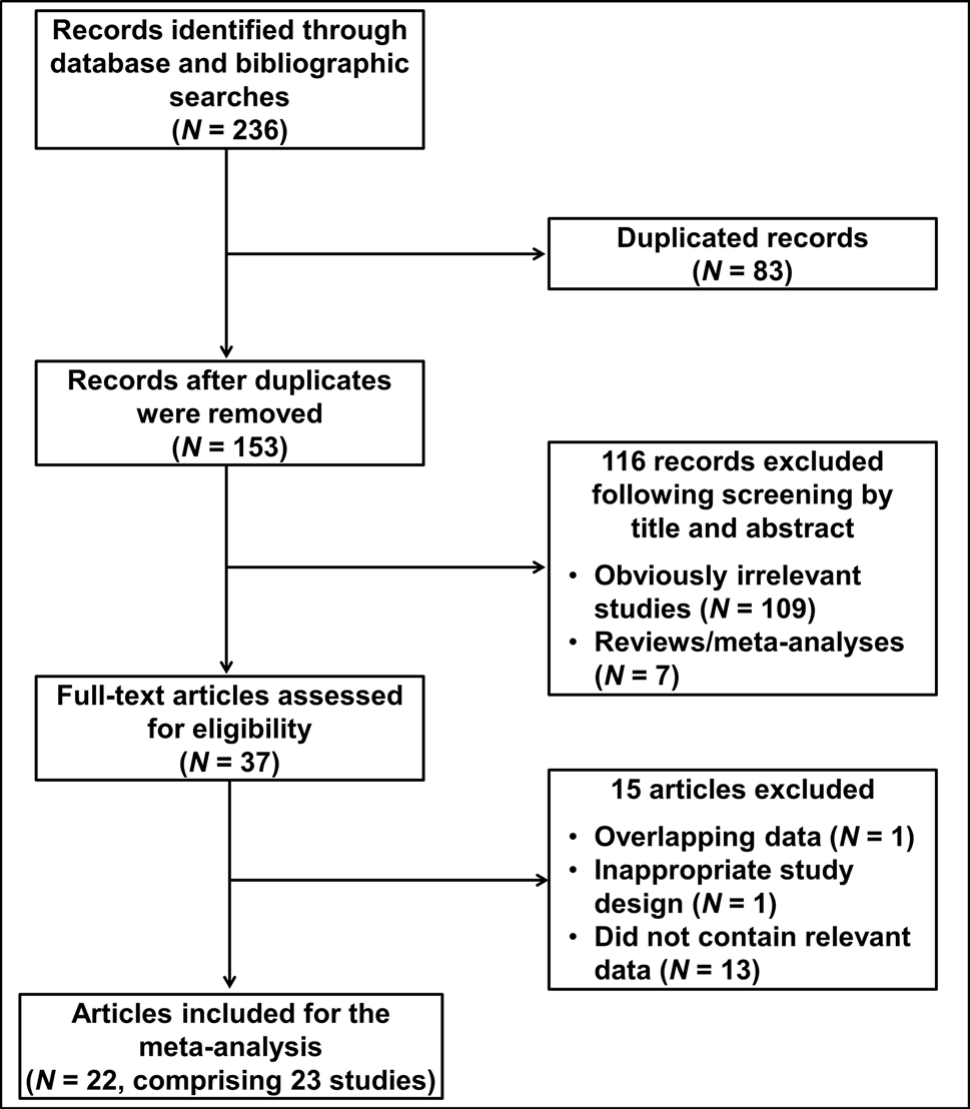
Flow diagram of study selection.

The study selection process is depicted in Fig. 1. The initial database and bibliographic searches identified 236 records (PubMed, N = 51; Scopus, N = 44; WoS, N = 114; Wanfang, N = 20; CNKI, N = 7; VIP, N = 0). After duplicated records were removed, 153 articles were screened by title and abstract. Thirty seven (37) articles were subsequently identified as potentially relevant and checked for eligibility by full-text review. Of these, 13 articles that did not meet the eligibility criteria and one article that had an inappropriate study design (as male controls were included in the analysis)^30^ were excluded. In addition, two articles were found to contain overlapping data^18,31^, and the one with the smaller sample size was excluded^31^. Ultimately, 22 articles comprising 23 studies were included for the quantitative synthesis of data^3,18,21,24,25,27,32–47^.

The 23 included studies involved a total of 34,721 subjects (12,766 cases and 21,955 controls). Among the included studies, eight (from seven articles) reported data for pre- and postmenopausal women separately^3,18,21,25,34,38,41^, and four other studies included only postmenopausal women^24,27,39,40^. The remaining studies either did not mention the menopausal status or did not perform separate analyses for pre- and postmenopausal women. In terms of ethnicity, nine studies were conducted on Asians^3,18,25,32,34,37,38,41,44^, nine on Caucasian^21,24,27,33,35,36,39,40,47^, three on other ethnicities^43,45,46^, and two on mixed ethnicities^21,42^. All studies were case–control in design. Fifteen (15) of the studies were considered as having high quality, whereas eight had low quality (Supplementary Table S1 online). The characteristics of the included studies are summarized in Table 1.

**Table 1:** Characteristics of the included studies.

Study ID^references^	Country	Ethnicity	Genotyping method	Cases			Controls			HWE P-value (controls)
				AA	AG	GG	AA	AG	GG	
Carrillo-Moreno 2019^43^	Mexico	Other	PCR–RFLP	245	175	42	158	145	31	0.784
Dai 2019^44^	China	Asian	MassARRAY	289	144	26	349	179	21	0.742
Sierra‑Martínez 2018^45^	Mexico	Other	Taqman	55	24	17	59	39	6	0.894
Atoum 2017^46^	Jordan	Other	PCR–RFLP	46	71	39	51	82	9	0.002
Madeira 2014^47^	Brazil	Caucasian	PCR–RFLP	5	47	12	0	58	14	< 0.001
Lu 2014^32^	China	Asian	PCR–RFLP	363	158	21	623	332	61	0.063
Ramalhinho 2013^33^	Portugal	Caucasian	PCR–RFLP	35	47	25	55	59	7	0.084
Javed 2011^25^	Pakistan	Asian	PCR–RFLP	38	47	12	40	39	20	0.076
Sakoda 2011^34^	China	Asian	SNaPshot	395	197	22	569	277	30	0.600
Dunning 2009^35^	UK	Caucasian	Taqman	1682	1967	521	1873	2048	526	0.347
González-Zuloeta Ladd 2008^24^	Netherlands	Caucasian	PCR–RFLP	72	94	24	1602	1648	453	0.359
Hu 2007^3^	China	Asian	Sequencing	76	34	3	68	35	7	0.395
Slattery 2007 (non-Hispanic)^21^	USA	Caucasian	PCR–RFLP	492	528	143	564	600	164	0.821
Slattery 2007 (mixed)^21^	USA	Mixed	PCR–RFLP	287	235	52	351	313	61	0.452
Wang 2007^36^	USA	Caucasian	PCR–RFLP	178	176	38	315	365	108	0.890
Shen 2006^37^	China	Asian	PCR–RFLP	149	84	14	168	87	21	0.046
Lu 2005^38^	China	Asian	PCR–RFLP	84	48	6	65	69	6	0.019
Onland-Moret 2005^39^	Netherlands	Caucasian	PCR–RFLP	122	130	55	123	151	61	0.223
Modugno 2005^40^	USA	Caucasian	PCR–RFLP	26	112	109	482	1822	1631	0.438
Wedrén 2004^27^	Sweden	Caucasian	Minisequencing	588	560	143	577	610	161	0.991
Cai 2003^41^	China	Asian	PCR–RFLP	536	497	36	610	508	49	< 0.001
Comings 2003^42^	USA	Mixed	PCR-RELP	22	35	10	62	64	19	0.699
Shin 2003^18^	Korea	Asian	PCR–RFLP	130	60	11	86	102	7	< 0.001

*HWE* Hardy–Weinberg equilibrium, *PCR–RFLP* polymerase chain reaction-restriction fragment length polymorphism.

### Meta-analysis results

**Figure 2:**
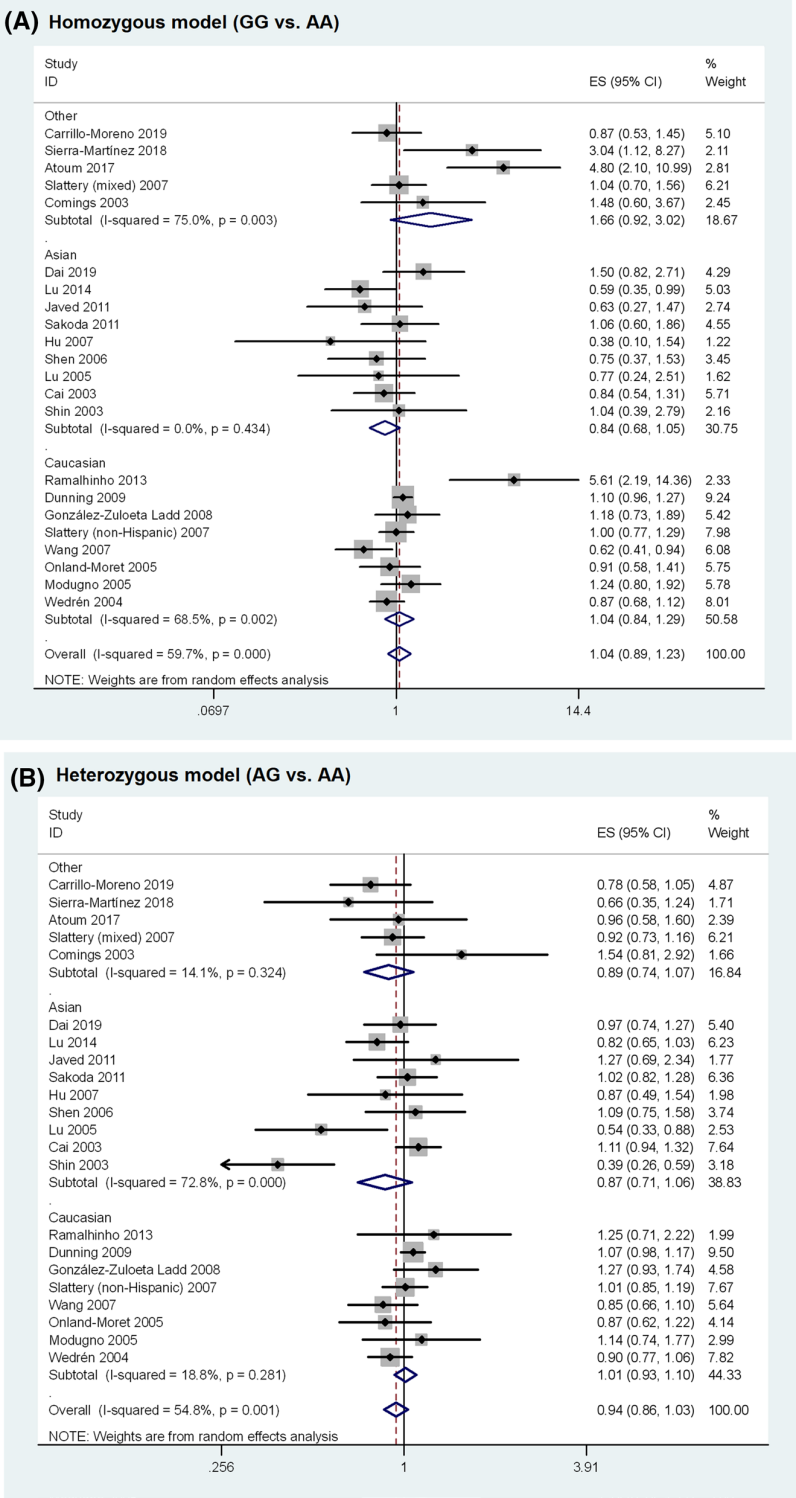

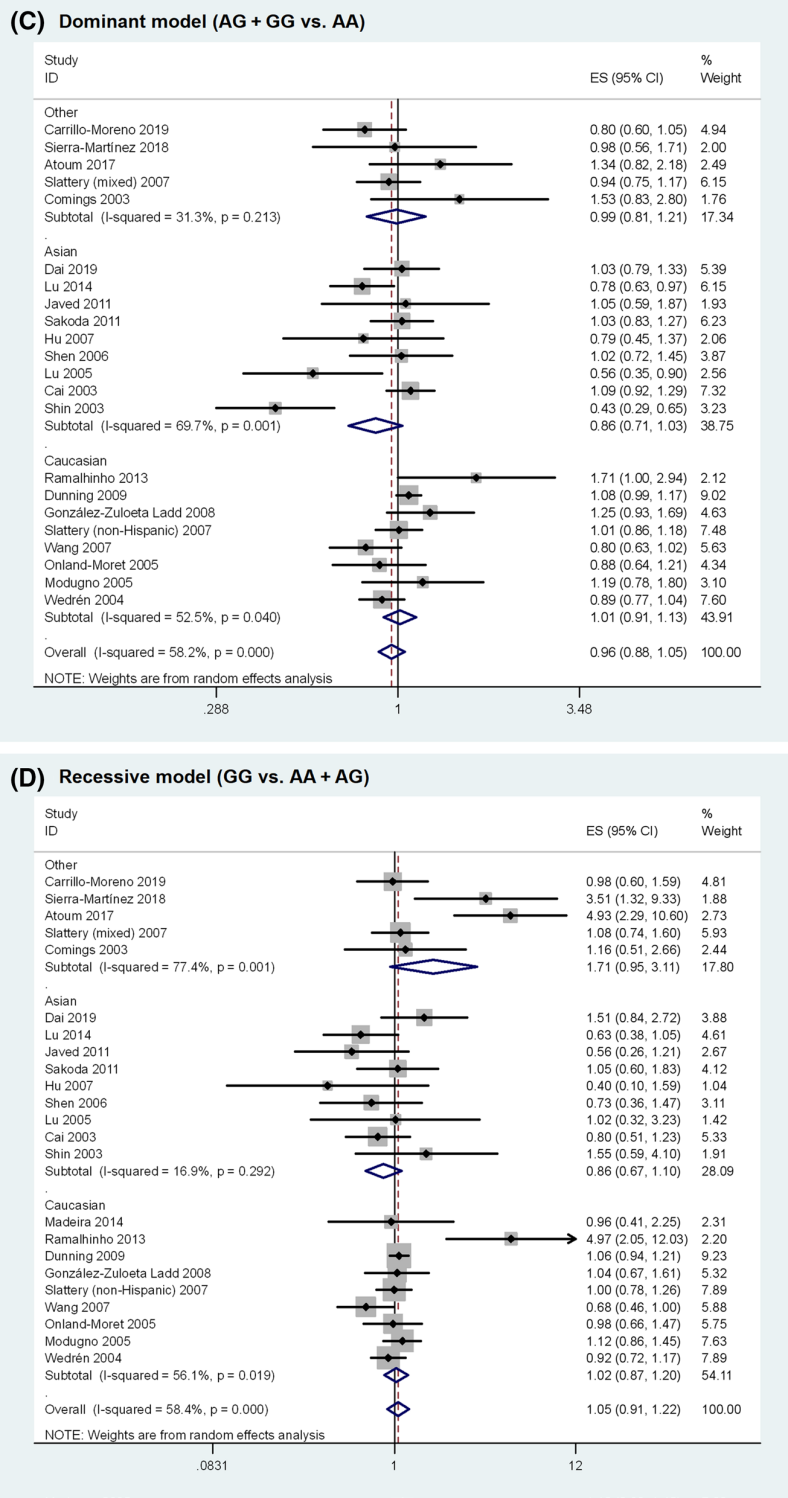

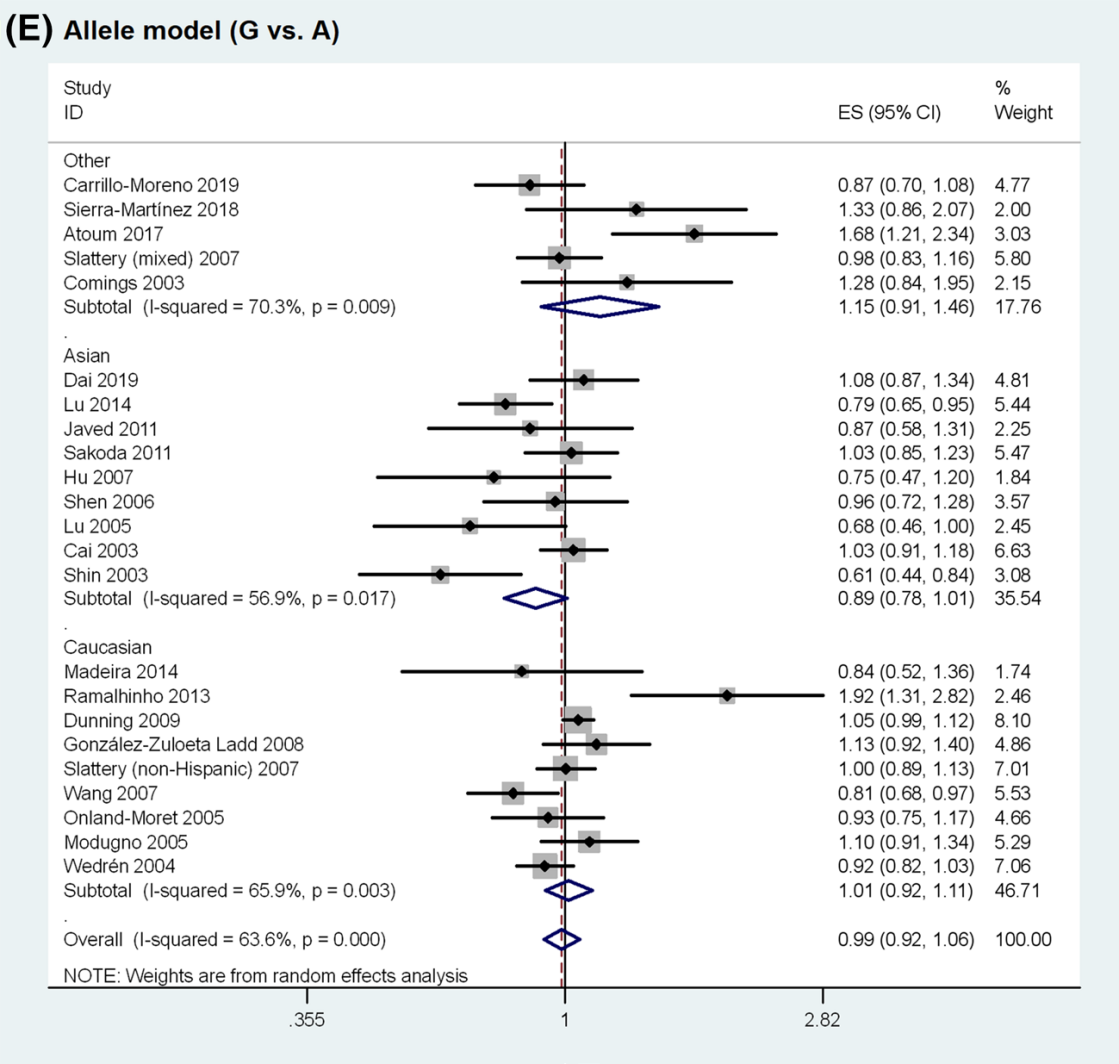
Forest plots of the association between *ESR1* rs9340799 polymorphism and breast cancer susceptibility.

The meta-analysis results are shown in Table 2. Overall, no statistically significant association was observed between *ESR1* rs9340799 polymorphism and BC susceptibility (homozygous model, OR = 1.045, 95% CI 0.887–1.231, P = 0.601; heterozygous model, OR = 0.941, 95% CI 0.861–1.030, P = 0.186; dominant model, OR = 0.957, 95% CI 0.875–1.045, P = 0.327; recessive model, OR = 1.053, 95% CI 0.908–1.222, P = 0.495; allele model, OR = 0.987, 95% CI 0.919–1.059, P = 0.709). The random-effects model was used in the above analyses as significant heterogeneity was present in all genetic models. The forest plots of the associations are presented in Fig. 2. Sensitivity analysis revealed that none of the individual studies had significant impact on the pooled OR (Supplementary Fig. S1 online).

**Table 2:** Summary of the association between *ESR1* rs9340799 polymorphism and breast cancer susceptibility.

Comparison model	No. of studies	No. of cases	No. of controls	Effect model	OR (95% CI)*	P-value
**Homozygous model**						
Overall	23	7296	12,323	Random	1.045 (0.887–1.231)	0.601
Asian	9	2211	2800	Fixed	0.845 (0.677–1.054)	0.135
Caucasian	9	4270	8716	Random	1.042 (0.845–1.285)	0.701
Premenopause	6	788	980	Fixed	0.825 (0.561–1.213)	0.327
Postmenopause	10	1697	5663	Fixed	0.970 (0.819–1.149)	0.725
High quality	15	5593	10,581	Random	1.109 (0.903–1.362)	0.322
Low quality	8	1703	1742	Fixed	0.921 (0.741–1.145)	0.461
**Heterozygous model**						
Overall	23	11,385	18,482	Random	0.941 (0.861–1.030)	0.186
Asian	9	3329	4206	Random	0.866 (0.707–1.061)	0.164
Caucasian	9	6861	12,952	Fixed	1.020 (0.956–1.089)	0.546
Premenopause	6	1294	1558	Random	0.891 (0.682–1.163)	0.396
Postmenopause	10	2547	7921	Random	0.908 (0.734–1.124)	0.375
High quality	15	8713	15,674	Fixed	0.998 (0.942–1.057)	0.939
Low quality	8	2672	2808	Random	0.851 (0.673–1.075)	0.176
**Dominant model**						
Overall	23	12,766	21,955	Random	0.957 (0.875–1.045)	0.327
Asian	9	3480	4428	Random	0.857 (0.713–1.031)	0.102
Caucasian	9	7931	16,077	Random	1.011 (0.905–1.130)	0.842
Premenopause	6	1345	1637	Random	0.812 (0.557–1.183)	0.278
Postmenopause	12	4000	11,631	Random	0.915 (0.785–1.066)	0.253
High quality	15	9907	18,945	Random	0.991 (0.897–1.095)	0.861
Low quality	8	2859	3010	Random	0.869 (0.714–1.058)	0.162
**Recessive model**						
Overall	23	12,766	21,955	Random	1.053 (0.908–1.222)	0.495
Asian	9	3480	4428	Fixed	0.858 (0.690–1.066)	0.166
Caucasian	9	7931	16,077	Random	1.023 (0.872–1.200)	0.784
Premenopause	6	1345	1637	Fixed	0.812 (0.557–1.183)	0.278
Postmenopause	10	2917	10,265	Fixed	1.003 (0.868–1.158)	0.967
High quality	15	9907	18,945	Random	1.091 (0.916–1.299)	0.330
Low quality	8	2859	3010	Fixed	0.954 (0.773–1.179)	0.664
**Allele model**						
Overall	23	12,766	21,955	Random	0.987 (0.919–1.059)	0.709
Asian	9	3480	4428	Random	0.888 (0.782–1.009)	0.068
Caucasian	9	7931	16,077	Random	1.015 (0.924–1.114)	0.760
Premenopause	6	1345	1637	Fixed	0.952 (0.845–1.074)	0.426
Postmenopause	10	2917	10,265	Fixed	0.972 (0.904–1.046)	0.451
High quality	15	9907	18,945	Random	1.018 (0.933–1.111)	0.687
Low quality	8	2859	3010	Fixed	0.949 (0.875–1.030)	0.212

*OR odds ratio, *CI* confidence interval.

### Subgroup analyses

Subgroup analyses were performed based on the ethnicity (Asian vs. Caucasian) and menopausal status (premenopause vs. postmenopause) of the study subjects, as well as the quality of the studies (high quality vs. low quality). No statistical significant association was observed for all subgroups under all genetic models (P > 0.05; Table 2).

Although significant heterogeneity was observed in the overall analysis, several subgroups were found to have low heterogeneity based on the *I*^2^ value. In the homozygous model, low heterogeneity was found for Asians (*I*^2^ = 0.0%), premenopause (*I*^2^ = 0.0%), postmenopause (*I*^2^ = 0.0%) and low quality (*I*^2^ = 19.8%) subgroups. A similar observation was observed for the recessive model (Asians, *I*^2^ = 16.9%; premenopause, *I*^2^ = 0.0%; postmenopause, *I*^2^ = 0.0%; low quality, *I*^2^ = 45.4%). In heterozygous model, the Caucasian (*I*^2^ = 18.8%) and high quality (*I*^2^ = 34.9%) subgroups showed low heterogeneity, whereas in allele model, low heterogeneity was noted in premenopause (*I*^2^ = 24.5%), postmenopause (*I*^2^ = 47.5%) and low quality subgroups (*I*^2^ = 46.7%). All subgroups in the dominant model showed high heterogeneity (*I*^2^ > 50%).

### Publication bias

**Figure 3:**
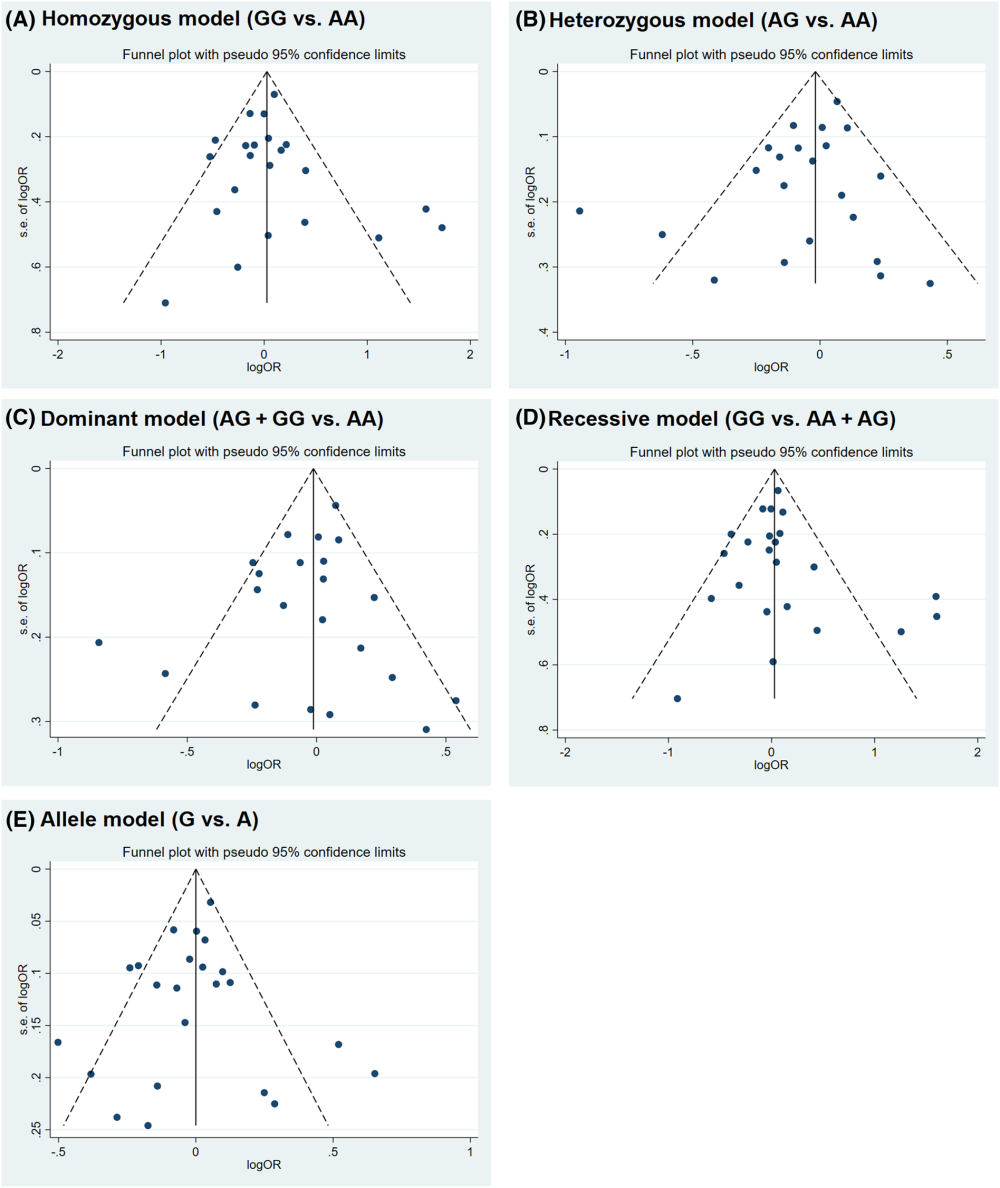
Funnel plots for assessing publication bias.

No evidence of asymmetry was detected in the funnel plots of all genetic models (Fig. 3), indicating the absence of publication bias. This observation was corroborated by the results of Begg’s and Egger’s tests (homozygous model, Begg's test P = 0.529, Egger's test P = 0.625; heterozygous model, Begg's test P = 0.978, Egger's test P = 0.152; dominant model, Begg's test P = 0.800, Egger's test P = 0.366; recessive model, Begg's test P = 0.488, Egger's test P = 0.303; allele model, Begg's test P = 0.636, Egger's test P = 0.937).

## Discussion

ERα, a member of the nuclear receptor superfamily, is encoded by a ~ 300 kb gene, *ESR1*, which is mapped to chromosomal locus 6q25.1 and contains eight exons. It has been documented that the human *ESR1* gene contains at least nine promoters, whereby each promoter harbors multiple transcription factors-binding sites^48^. The ERα protein possesses DNA- and ligand-binding domains which are highly conserved^49^. It is depicted that ERα can mediate the effect of estrogen via several molecular pathways. Among these, the classical pathway is the best-known. In this direct pathway, unliganded ERα forms a cytosolic complex with Hsp90. Upon estrogen binding to the ligand-binding domains of ERα, the ERα-Hsp90 complex dissociates. Subsequently, ERα dimerizes and translocates to the nucleus. Following that, the DNA-binding domains of ERα, consisting of two functionally distinct zinc finger motifs, bind to a characteristic stretch of DNA sequence named the estrogen response elements in the promoters of the target genes to influence the process of transcription^50^.

Meanwhile, the tethered pathway entails protein–protein interaction or heterodimerization of ERα with other transcription factors such as AP1or NF-kB after ligand activation. This results in the indirect binding of DNA by ERα, contributing to the regulation of target genes including insulin-like growth factor 1, cathepsin D, progesterone receptor, transforming growth factor α, pS2, retinoic acid receptor α1, c-myc, etc., which are essential for cell proliferation and survival^51^. The nongenomic pathway typically involves a small plasma membrane population- and cytoplasm-based ERα^52^, which interacts with signaling proteins such as Src, mitogen-activated protein kinase (MAPK) and phosphoinositide 3-kinase. These signaling molecules can activate the phosphorylation of ERα and its coregulators^53,54^. This subsequently triggers signaling cascades via second messengers (SM), and eventually, it enhances nuclear ERα signaling without involving gene regulation. The last ER pathway is the ligand-independent pathway. In this case, ERs can become activated via crosstalk with other signaling pathways, e.g. the insulin-like growth factor-1 receptor and the epidermal growth factor receptor pathways^55^. In these instances, ERs are activated by phosphorylation to form dimers, to bind DNA, and regulate the expression of genes.

Notwithstanding, all models of ERα signaling pathways point to the vital role of ERα in the proliferation and survival of breast epithelial cells, as well as mammary tumorigenesis^54^. ER has been used as a molecular classifier for breast tumors, whereby BC can be graded as ER-positive and ER-negative. A large proportion (~ 75%) of BC are known to be ER-positive^56^. ERα-positive cases are often associated with more optimistic prognosis as they generally respond more positively to endocrine therapies, and are also sensitive to CDK4/6 inhibitors^56,57^. In contrast, ERα-negative BC is generally regarded as aggressive and metastatic malignancies^58^.

Given the important role of ERα in BC, its level and structure need to be tightly regulated to ensure an optimal functionality. The level and structure of a protein are known to be influenced by, among others, genetic polymorphisms^59^. For this reason, many genetic association studies have investigated the relationship between *ESR1* polymorphisms and BC susceptibility. These polymorphisms include, but not limited to, rs9340799, ﻿rs3020364, rs9322335, ﻿rs2234693, rs1801132, rs2046210, rs3020314, rs1514348, rs3020314, rs1514348, rs1514348 and ﻿rs3020314^35,60–63^.

Among these many polymorphisms, we have chosen to focus on rs9340799, an intronic polymorphism located just upstream of exon 2 of *ESR1*. This is because the rs9340799 polymorphism has been widely studied and conflicting results have been frequently obtained, and no recent meta-analysis has been carried out to address the inconsistencies in the study findings. For instance, while Wang et al. reported that the GG genotype of the polymorphism was associated with a reduced susceptibility to BC, Sierra‑Martínez et al. reported that the same genotype was associated with an increased susceptibility to BC^36,45^. Besides, Sakoda et al. did not find any significant association between the polymorphism and BC susceptibility^34^. The difference in the study findings could be attributed to the variations in allele frequency across different studies. These variations are particularly relevant in populations consisting of different ethnicities, as interethnic differences in allele frequencies have long been known^64,65^. Taking the examples above, while Wang et al.^36^ noted in a Caucasian population that the minor allele frequency (MAF) of the polymorphism was 0.369, Sakoda et al.^34^ found that the MAF was merely 0.192 in an Asian population. These variations can account for differences in gene expression and therefore, disease susceptibility^66,67^. It is thus important to take into account the population variations in the allele frequency when attempting to identify a genetic biomarker for early identification of a disease^68^. For this reason, heterogeneity tests and subgroup analysis by ethnicity need to be performed when pooling the results from different studies together, as were done in our meta-analysis.

It is noteworthy that most of these studies have centered on genetic association rather than deciphering the exact biological mechanisms. Nonetheless, it has been postulated that intronic polymorphisms such as the rs9340799 polymorphism of *ESR1* may influence the cancer susceptibility by (i) being in linkage disequilibrium with another functional polymorphism in the same locus; (ii) influencing the expression of other genes through alterations to their transcriptional activity or mRNA stability; (iii) containing regulatory sequences which can impact gene expression via transcriptional regulation^47,69^. For these reasons, in this meta-analysis, we attempted to precisely re-examine the relationship between the *ESR1* rs9340799 polymorphism and the susceptibility to BC. In doing so, we included 23 case–control studies from 22 systematically selected published articles. We performed the meta-analysis under five different genetic models, namely the homozygous, heterozygous, dominant, recessive, and allele models. Importantly, our analyses with all five genetic models failed to detect any significant association between the rs9340799 polymorphism and BC susceptibility. Under each genetic model, we further stratified our analysis based on the following subgroups: (i) ethnicity (Asian vs. Caucasian), (ii) menopausal status (premenopause vs. postmenopause), and (iii) study quality (high quality vs. low quality). Again, none of these subgroups showed any significant association. Notably, our finding was in agreement with that of the Zhang et al. even though we have included more studies (N = 23 vs. N = 7)^29^.

The major strength of our study is that we have analyzed data from a large population of meticulously selected studies; therefore, this study has strong statistical power. Besides, the chosen exposure, i.e., the rs9340799 polymorphism, is a discrete and well-defined parameter that can be genotyped with high precision using the available technologies. This allows a fair comparison to be made among independent studies, contributing to more consistent inter-laboratory or inter-study comparison. On the other hand, the major limitation of this study is that gene–gene or gene-environment interactions were not measured as most of the included studies did not report this information. Furthermore, our meta-analysis has so far focused on one polymorphism from *ESR1*. The analyses of more polymorphisms of *ESR1* in future, either individually or in tandem, may further reveal the synergistic effects of such polymorphisms in influencing BC susceptibility^70^.

In conclusion, our overall results revealed no significant association between the rs9340799 polymorphism of *ESR1* and the susceptibility to BC, despite the different genetic models considered. Each genetic model was further divided into subgroups based on ethnicity, study quality and menopausal status, but similarly, no statistically significant association was observed. Nevertheless, our conclusion warrants further studies, given that the *ESR1* harbors many polymorphisms that await detailed investigation.

## Methods

### Literature search

A comprehensive literature search was performed in the Web of Science (WoS), PubMed, Scopus, China National Knowledge Infrastructure (CNKI), VIP and Wanfang databases up to January 21st, 2021, without language restriction. The following search terms were used: (ESR1 OR estrogen receptor) AND (XbaI OR rs9340799) AND (polymorphism or variant) AND (breast cancer OR breast neoplasm). Studies were selected if they fulfilled the following inclusion criteria: (i) were case–control and/or cohort studies which have investigated the association between *ESR1* rs9340799 polymorphism and BC susceptibility, and (ii) reported the genotype and allele frequencies or contained necessary data to obtain the information. Studies were excluded if (i) they were not original research papers (e.g. review articles or commentaries), and (ii) the investigations were not performed on human subjects. The reference lists of the eligible studies were also manually screened to identify additional relevant studies. When overlapping data were found, we included only the study with the largest sample size. The study protocol was pre-registered with PROSPERO (registration number: CRD42021231912).

### Data extraction and quality assessment

Three investigators independently extracted the following data from the included studies: name of the first author, publication year, location, ethnic group, sample size, genotype and allele frequencies, menopausal status, genotyping method, blinding status, genotyping success rate, and sources of controls. Discrepancies were resolved through discussion until a mutual agreement was reached. The P-values of the Hardy–Weinberg equilibrium (HWE) among the control group was calculated using a goodness-of-fit test. The Modified Newcastle–Ottawa Scale for Case–Control Studies of Genetic Association was used to assess the quality of the included studies^71^. Studies rated ≥ 6 stars were considered high quality.

### Statistical analysis

STATA version 16.0 (StataCorp, College Station, Texas, USA) was used for the quantitative synthesis of the data. The association between *ESR1* rs9340799 polymorphism and BC susceptibility was evaluated using the odds ratio (OR) for various genetic models, i.e. homozygous (GG vs. AA), heterozygous (AG vs. AA), dominant (AG + GG vs. AA), recessive (GG vs. AA + AG) and allele (G vs. A). A forest plot was also generated to graphically represent the findings. A fixed-effect model was used if the heterogeneity among the studies was low (Cochran’s Q P-value of > 0.1 and *I*^2^ value of < 50%). On the other hand, when heterogeneity was significant, a random-effects model was used. Sensitivity analysis was performed using the leave-one-out method for evaluating the robustness of the findings. Subgroup analyses were performed according to ethnicity (Asian vs. Caucasian), study quality (high quality vs. low quality), and menopausal status (premenopause vs. postmenopause). In most included studies, the ethnicity was explicitly stated, although the standards of classification (i.e. self-reported or via genetic analyses) was not known. However, when such information was not available, the populations were classified into different ethnicities based on the major ethnic group of the countries in which the subjects were recruited. Publication bias was evaluated using the Begg’s and the Egger’s tests, and through visual inspection of the funnel plot for asymmetry. For all analyses, the result was considered to be statistically significant when P < 0.05, unless otherwise stated.

## Supplementary Information

Supplementary Information.
