# Supplementary material for: The rs9340799 polymorphism of the estrogen receptor alpha (ESR1) gene and its association with breast cancer susceptibility
Source: Sci Rep. 2021 Sep 20;11:18619. doi: 10.1038/s41598-021-97935-8 (PMC8452701; doi:10.1038/s41598-021-97935-8)
Supplement: Supplementary file 1 — Supplementary Information. [file 41598_2021_97935_MOESM1_ESM.docx]

**Supplementary Table S1**. Quality assessment of the included studies

| **Study ID^references^** | **Selection** | | | | **Comparability** | **Exposure** | | | **Total star** |
| --- | --- | --- | --- | --- | --- | --- | --- | --- | --- |
|  | **Criterion 1** | **Criterion 2** | **Criterion 3** | **Criterion 4** | **Criterion 1** | **Criterion 1** | **Criterion 2** | **Criterion 3** |  |
| Carrillo-Moreno 2019^32^ | ★ | ★ |  | ★ | ★ |  | ★ |  | 5 |
| Dai 2019^33^ | ★ | ★ |  | ★ | ★★ |  | ★ | ★ | 7 |
| Sierra‑Martínez 2018^34^ | ★ | ★ |  | ★ | ★ |  | ★ |  | 5 |
| Atoum 2017^35^ | ★ | ★ |  | ★ | ★ |  | ★ | ★ | 6 |
| Madeira 2014^36^ | ★ | ★ |  | ★ | ★ |  | ★ | ★ | 6 |
| Lu 2014^37^ | ★ | ★ | ★ | ★ | ★ |  | ★ | ★ | 7 |
| Ramalhinho 2013^38^ | ★ | ★ |  | ★ | ★ |  | ★ | ★ | 6 |
| Javed 2011^25^ |  | ★ |  |  | ★ |  | ★ | ★ | 4 |
| Sakoda 2011^39^ | ★ | ★ | ★ | ★ | ★ |  | ★ |  | 6 |
| Dunning 2009^40^ | ★ | ★ | ★ | ★ | ★ |  | ★ |  | 6 |
| González-Zuloeta Ladd 2008^24^ | ★ | ★ | ★ |  | ★★ |  | ★ | ★ | 7 |
| Hu 2007^41^ | ★ | ★ |  | ★ | ★ |  | ★ |  | 5 |
| Slattery 2007 (non-Hispanic)^21^ | ★ | ★ | ★ |  | ★★ |  | ★ |  | 6 |
| Slattery 2007 (mixed)^21^ | ★ | ★ | ★ |  |  |  | ★ |  | 4 |
| Wang 2007^42^ | ★ | ★ | ★ | ★ | ★★ |  | ★ | ★ | 8 |
| Shen 2006^43^ | ★ | ★ |  | ★ | ★ |  | ★ |  | 5 |
| Lu 2005^44^ | ★ | ★ |  | ★ | ★★ |  | ★ | ★ | 7 |
| Onland-Moret 2005^45^ | ★ | ★ | ★ | ★ | ★ |  | ★ |  | 6 |
| Modugno 2005^46^ | ★ | ★ | ★ |  | ★★ |  | ★ |  | 6 |
| Wedrén 2004^27^ | ★ | ★ | ★ |  | ★ |  | ★ | ★ | 6 |
| Cai 2003^47^ | ★ | ★ |  | ★ | ★ |  | ★ |  | 5 |
| Comings 2003^48^ | ★ | ★ | ★ | ★ | ★ |  | ★ | ★ | 7 |
| Shin 2003^18^ | ★ | ★ |  | ★ | ★★ |  |  |  | 5 |

* Quality assessment was done by using the Modified Newcastle-Ottawa Scale for Case-Control Studies of Genetic Association ^67^. Studies with ≥6 stars were considered to be of high quality.


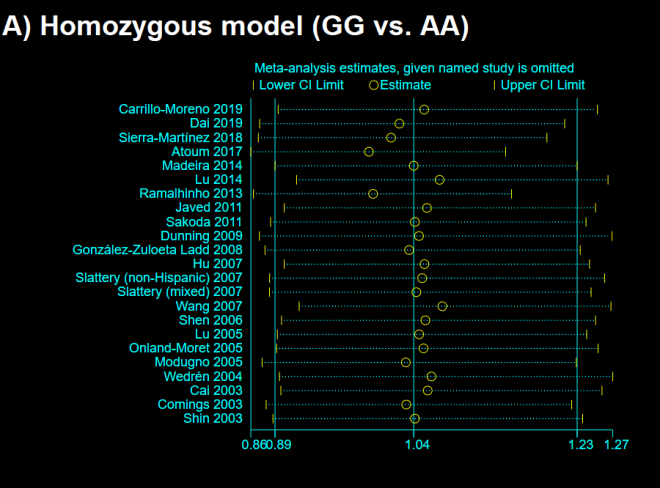

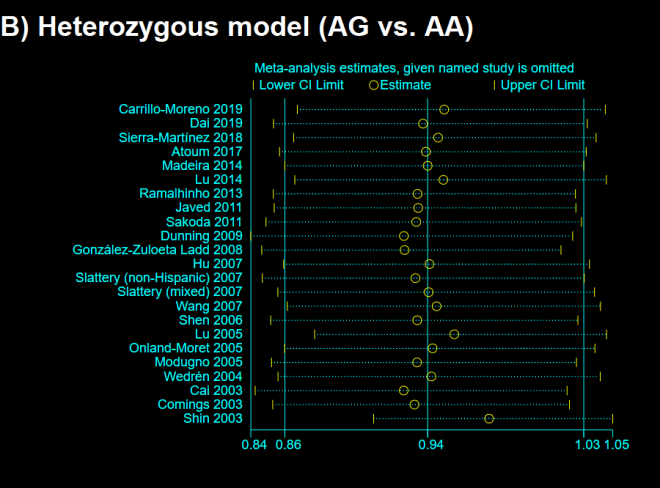

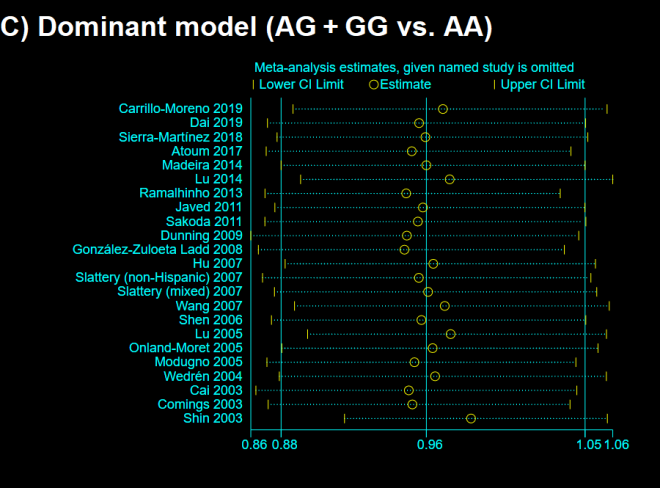

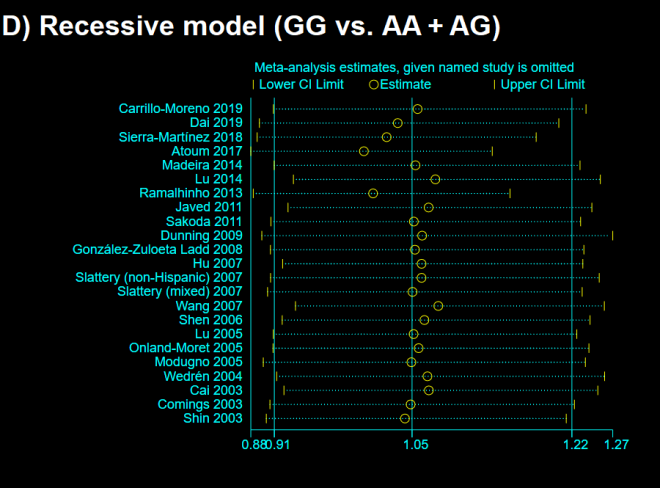

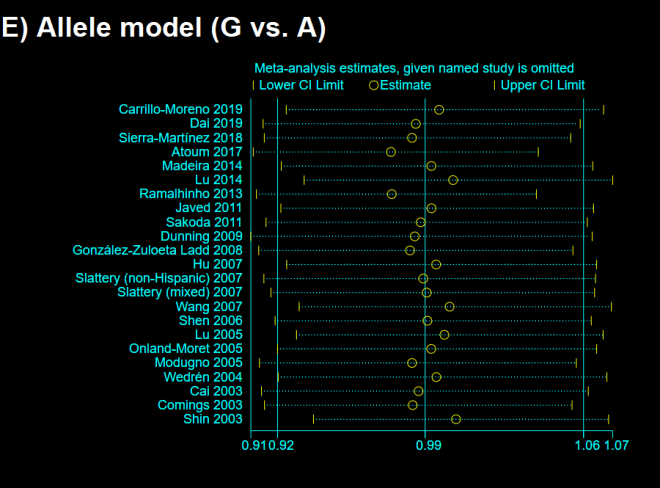


**Supplementary Figure S1**. Sensitivity analysis of the association between *ESR1* rs9340799 polymorphism and breast cancer susceptibility
